# 5-methoxyresorcinol mitigates postmenopausal osteoporosis through regulation of the PI3K–AKT–GSK3β signaling pathway and ROS homeostasis

**DOI:** 10.3389/fphar.2026.1734275

**Published:** 2026-05-18

**Authors:** Maosheng Yang, Rongrong Li, Hanbin Wang, Yitong Geng, Zhiyuan Yv, Jinqiao Qiao, Yi Li

**Affiliations:** Department of Joint Surgery and Sports Medicine, Shandong Provincial Hospital Affiliated to Shandong First Medical University, Jinan, China

**Keywords:** 5-methoxyresorcinol, network pharmacology, osteoclast, osteoporosis, PI3K-Akt signaling pathway, reactive oxygen species

## Abstract

**Objectives:**

Osteoporosis is a systemic skeletal disease characterized by excessive osteoclast-mediated bone resorption. This study aimed to evaluate the therapeutic potential of 5-methoxyresorcinol (MR) in osteoporosis and to clarify its underlying mechanisms.

**Methods:**

Network pharmacology analysis was performed to identify shared targets between MR and osteoporosis. *In vitro* experiments using bone marrow-derived macrophages were performed to assess the effects of MR on osteoclast differentiation and function. In addition, the *in vivo* efficacy of MR was evaluated in an ovariectomized (OVX) mouse model using micro-CT and histological analyses.

**Results:**

Network pharmacology analysis identified 107 potential targets of MR and 8,060 osteoporosis-associated genes, of which 50 were overlapping targets. Among these targets, the PI3K–AKT signaling pathway showed significant enrichment. *In vitro*, MR at concentrations ≤1 mM showed no significant cytotoxicity and suppressed osteoclast differentiation in a dose-dependent manner. MR also inhibited osteoclast function by reducing the formation of F-actin ring and the secretion of acidified vesicles. Mechanistically, MR inhibited activation of the PI3K–AKT–GSK3β pathway, downregulated osteoclast-specific genes (DC-STAMP, OC-STAMP, CTSK, MMP-9, c-FOS, and NFATc1), and reduced RANKL-induced reactive oxygen species (ROS) generation. In OVX mice, MR improved bone microarchitecture and alleviated osteoporotic bone loss.

**Conclusion:**

MR attenuates osteoporosis by suppressing osteoclast differentiation and function through regulating PI3K–AKT–GSK3β axis and ROS homeostasis, supporting its potential as a promisingtherapeutic candidate for osteoporosis.

## Graphical Abstract

**Figure:**
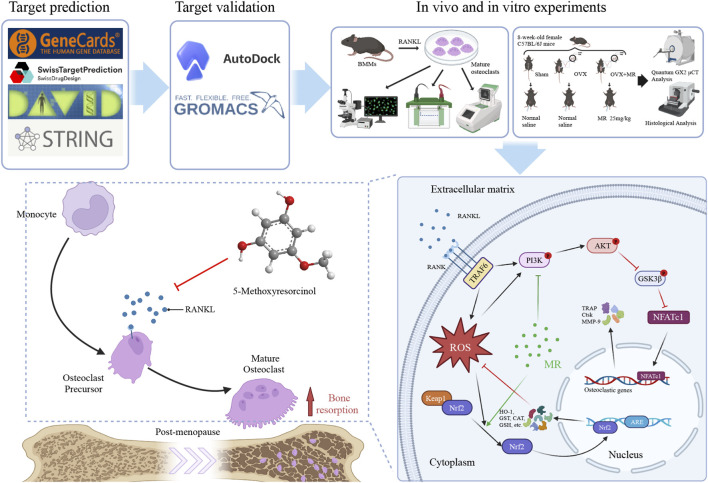

## 1 Introduction

Osteoporosis is a systemic metabolic bone disorder characterized by reduced bone mass and deterioration of bone microarchitecture, ultimately resulting in increased bone fragility and fracture risk (Arceo-Mendoza and Camacho, 2015; Morin et al., 2025). With the progressive aging of the global population, the incidence of osteoporosis has risen substantially in recent decades. It is estimated to affect more than 200 million people worldwide and to account for nearly 9 million fractures each year, with a substantially higher prevalence in women (Geiker et al., 2020; GBD 2019 Fracture Collaborators, 2021). Pathophysiologically, osteoporosis is primarily characterized by an imbalance in bone remodeling, where bone resorption by osteoclasts consistently exceeds bone formation by osteoblasts (Huang et al., 2020; Manolagas, 2000).

Osteoclasts are multinucleated cells derived from hematopoietic precursors and are pivotal in both physiological bone remodeling and pathological bone loss (Elson et al., 2022). Osteoclast proliferation, differentiation, and maturation are tightly regulated by two critical cytokines, receptor activator of nuclear factor-κB ligand (RANKL) and macrophage colony-stimulating factor (M-CSF) (Boyle et al., 2003; Okamoto et al., 2017). RANKL, a component of the TNF family of cytokines, is produced mainly by osteoblasts and stromal cells in either membrane-bound or secreted forms. It binds to the RANK (receptor activator of nuclear factor-κB), which is highly expressed on osteoclast surfaces (Matsumoto et al., 2017). That interaction recruits TRAF6 (tumor necrosis factor receptor-associated factor 6) (Okamoto et al., 2017), thereby activating downstream signaling cascades, including nuclear factor-κB (NF-κB), phosphatidylinositol 3-kinase-protein kinase B (PI3K–AKT), and mitogen-activated protein kinase (MAPK) (Zou et al., 2021; Chen et al., 2020; Lee et al., 2021). These signaling events converge on NFATc1 (nuclear factor of activated T cells 1), a master transcription factor for osteoclastogenesis (Takayanagi et al., 2002), which subsequently drives the expression of osteoclast-related genes such as cathepsin K (CTSK), tartrate-resistant acid phosphatase (TRAP), dendritic cell-specific transmembrane protein (DC-STAMP), osteoclast stimulatory transmembrane protein (OC-STAMP), and matrix metalloproteinase 9 (MMP-9) (Matsuo et al., 2004; Kim et al., 2008; Miyamoto et al., 2012). Thus, targeting the RANKL axis has become a critical therapeutic strategy for inhibiting pathological osteoclastogenesis and treating osteoporosis.

Current pharmacological management of osteoporosis mainly includes antiresorptive agents (e.g., bisphosphonates, denosumab) and bone-forming agents (e.g., parathyroid hormone analogs, romosozumab) for osteoporosis treatment (Li et al., 2021; Lyu et al., 2020). However, these therapies have notable limitations, including poor patient adherence, rebound effects after discontinuation, and adverse effects such as medication-related atypical femoral fractures and jaw osteonecrosis (Modi et al., 2015; Cummings et al., 2018; Li et al., 2021). These limitations underscore the need to identify safer and more effective therapeuticstrategies for preventing disease progression and reducing the burden of osteoporosis.

Network pharmacology offers a powerful approach for elucidating the mechanisms of drug action by mapping complex interactions among drugs, signaling pathways, and disease phenotypes (Zhang et al., 2019). 5-methoxyresorcinol (MR, also known as Flamenol) is an active ingredient found in Pra-Sa-Chang-Dang, a traditional Thai herbal formula used for fever relief (Chaniad et al., 2024). Previous studies have suggested that this formula possesses multiple pharmacological activities, such as antiproliferative and anti-inflammatory effects (Prommee et al., 2022; Sireeratawong et al., 2012). Current study applied network pharmacology to predict the mechanistic link between MR and osteoporosis, thereby guiding experimental validation. The findings demonstrate that MR inhibiting RANKL-induced osteoclastogenesis and alleviates osteoporosis through inhibition of PI3K–AKT signaling pathway.

## 2 Materials and methods

### 2.1 Bioinformatics and network pharmacology analysis

Target genes linked to osteoporotic pathology were retrieved from the GeneCards database (link: http://www.genecards.org) using the keywords “*osteoporosis*” and “*osteoclasts.*” The canonical SMILES string of 5-methoxyresorcinol (PubChem CID: 71648) was retrieved from the PubChem database (link: https://pubchem.ncbi.nlm.nih.gov). Potential biological targets of the compound were predicted using the SwissTargetPrediction platform (link: http://swisstargetprediction.ch). Overlapping targets across datasets were identified using Venny (v2.1, link: https://bioinfogp.cnb.csic.es/tools/venny/). Gene Ontology (GO) functional annotation and Kyoto Encyclopedia of Genes and Genomes (KEGG) pathway enrichment analyses for the overlapping targets were performed using the DAVID bioinformatics resource (link: https://davidbioinformatics.nih.gov), restricting species to *Homo sapiens*. Protein–protein interaction (PPI) networks were generated through the STRING online platform (v12.0, link: https://cn.string-db.org) under the following parameters: 1) organism set to “*Homo sapiens*”; 2) minimum interaction score ≥0.4; and 3) automatic exclusion of disconnected nodes. Networks were visualized in Cytoscape (v3.10.0), and hub genes were screened according to Degree values.

### 2.2 Molecular docking

The 2D chemical structure of MR was obtained from PubChem. The 3D crystal structure of the PI3K protein target (PDB ID: 6DGT) was obtained from the Protein Data Bank (link: https://www.rcsb.org). Before docking, the receptor structure was prepared by removing water molecules and co-crystallized ligands and by adding hydrogen atoms. AutoDock Vina (version 1.5.6) was employed for molecular docking simulations with default settings. The binding conformations between MR and PI3K were subsequently analyzed and visualized using PyMOL Molecular Graphics System (version 2.6.2).

### 2.3 Molecular dynamics simulation

Molecular dynamics (MD) simulations were performed using GROMACS (v.2022). The ligand was parameterized using the Generalized Amber Force Field (GAFF), while the protein employed the AMBER ff14SB force field with TIP3P water solvation. After energy minimization, the protein–ligand complex was placed in a periodic boundary simulation box. Simulation parameters included LINCS-constrained hydrogen bonds (integration timestep: 2 fs) and Particle Mesh Ewald electrostatics (cutoff: 1.2 nm), with non-bonded interactions (10 Å cutoff) updated every 10 steps. System temperature and pressure were maintained at 298 K and 1 bar, respectively, using the V-rescale thermostat and Berendsen barostat. After sequential equilibration under NPT (100 ps) and NVT (100 ps) ensembles, production simulations were performed for 100 ns, recording conformational snapshots at 10 ps intervals. Trajectory analyses were conducted with VMD and PyMOL, and binding free energy was estimated using the MMPBSA method implemented in g_mmpbsa.

### 2.4 Cell culture, differentiation, and treatment

Primary bone marrow cells were isolated and cultured as previously described protocols (Meng et al., 2025; Yuan et al., 2024). Bone marrow was flushed from the tibias and femurs of C57BL/6J mice (Vital River Laboratories). All animal procedures were conducted in accordance with the guidelines of Shandong Provincial Hospital Animal Care Committee (Shandong First Medical University Affiliate; Protocol No. 2025-018). Cells were cultured in complete α-MEM containing 1% penicillin-streptomycin and 10% fetal bovine serum and at 37 °C in a humidified atmosphere containing 5% CO_2_ for 16–24 h. Non-adherent bone marrow-derived macrophages (BMMs) were then collected after erythrocyte lysis and sequential centrifugation. For expansion, cells were seeded at a density of 2 × 10^5^ cells/mL in complete medium containing 10 ng/mL M-CSF (576402, Biolegend, USA) and cultured for 48–72 h. To induce osteoclast differentiation, the culture medium was replaced with differentiation medium containing 30 ng/mL RANKL (769402, Biolegend, USA) and 10 ng/mL M-CSF for 4–6 days. Experimental groups were treated with DMSO-solubilized MR at concentrations of 0.5, 0.8, and 1 mM. For the rescue experiments, cells were treated with SC79 (MCE, HY-18749) at concentration of 2 μg/mL.

### 2.5 Cytotoxicity assay

In this study, we implemented CCK-8 assay (APExBIO, K1018) to assess the cytotoxic effects of MR on BMMs and osteoclast precursors. Cells were seeded in 96-well plates at a density of 1 × 10^4^ per well under standard culture conditions. After 48–72 h of pre-culture, the cells were exposed to differentiation medium containing escalating concentrations of the compound. Following 24 h of treatment, 10 μL of CCK-8 reagent was added to each well and the plates were incubated for an additional 2 h at 37 °C in the dark. Absorbance at 450 nm was measured using a TECAN SPARK microplate reader to assess cell viability.

### 2.6 TRAP staining

According to previous studies (Dong et al., 2024), osteoclast precursors were seeded in 24-well plates at a density of 2 × 10^5^ cells per well. On days 5–6 of differentiation, cells were fixed using 4% paraformaldehyde (PFA) at room temperature for 20 min and then washed three times with PBS. For preparation of the acetate-tartrate buffer, sodium tartrate (150 μg/mL), sodium acetate trihydrate (19 mg/mL), and glacial acetic acid (0.45%) were dissolved in distilled deionized water (ddH_2_O). Fast violet B salt (7 mg/mL) and naphthol AS-BI phosphate (2 mg/mL) solutions were separately dissolved in the buffer and filtered through 0.45 μm membranes. Then, mixed the above solution to produce the TRAP working solution. Cells were incubated in TRAP working solution at 37 °C for 120 min in the dark. We quantified TRAP^+^ multinucleated cells containing three or more nuclei from nine random fields per well using an Invitrogen EVOS M7000 microscope system (Thermo Fisher Scientific, USA).

### 2.7 F-actin staining

F-actin staining was carried out as previously reported (Meng et al., 2025). Mature osteoclasts were fixed with 4% PFA at room temperature for 20 min, then stained with Phalloidin-iFluor 594 (Abcam, ab176757) for 2 h in the dark. Following two PBS washes, nuclei were counterstained with DAPI for 5 min. F-actin ring structures were visualized using an Invitrogen EVOS M7000 system, with nine random fields captured per sample. Quantitative analysis was performed by determining the proportion of osteoclasts with intact actin sealing rings.

### 2.8 Acridine Orange staining

Acridine Orange (AO) staining was performed according to established protocols (Wang et al., 2024). On days 4–5 of osteoclast differentiation, cells in 24-well plates were incubated with 10 μg/mL AO (Sigma-Aldrich, A6014) in α-MEM medium at 37 °C for 15 min in the dark. After three washes with α-MEM, cells were immediately imaged using the EVOS M7000 imaging system. Fluorescence intensity was subsequently quantified using ImageJ software (v1.53 g).

### 2.9 Western blotting assay

Western blotting (WB) analysis was performed as described previously (Meng et al., 2025). Osteoclast precursors were washed with PBS and lysed in ice-cold RIPA buffer (Solarbio, R0010) containing 1% phosphatase inhibitor (Solarbio, P1260) and protease inhibitor (Solarbio, P6730). The lysates were centrifuged at 13,500 rpm for 20 min at 4 °C, and the supernatants were collected for protein quantification using a BCA assay kit (Solarbio, PC0020). Proteins (20 μg per sample) were denatured for 5 min at 95 °C, separated by SDS-PAGE (8%–10%), and transferred onto PVDF membranes (0.2 μm). Membranes were blocked with 5% non-fat milk at room temperature for 1 h and then incubated overnight at 4 °C with primary antibodies against NFATc1, AKT (phosphorylated/non-phosphorylated), PI3K (phosphorylated/non-phosphorylated), GSK3β (phosphorylated/non-phosphorylated), kelch-like ECH-associated protein 1 (Keap1), nuclear factor erythroid 2-related factor 2 (Nrf2), CTSK, MMP-9 and β-actin. Following incubation with HRP-conjugated secondary antibodies for 1–2 h, protein bands were detected using an enhanced chemiluminescence (ECL) substrate (Bio-Rad, 1705061). Band intensities were quantified using ImageJ software.

### 2.10 Quantitative real-time polymerase chain reaction

Total RNA was extracted using RNAex Pro reagent (Accurate Biology, AG21101), and complementary DNA (cDNA) was synthesized using the EVO M-MLV reverse transcriptase premix (Accurate Biology, AG11706). Quantitative real-time PCRwas performed on a LightCycler 480 II system (Roche Diagnostics) using SYBR Green chemistry (Accurate Biology, AG11701). Cycling parameters included the following steps: initial denaturation (95 °C, 30 s), thereafter 40 cycles of denaturation (95 °C, 5 s) and annealing/extension (60 °C, 30 s). A melting curve analysis was then performed with continuous heating from 60 °C to 95 °C, followed by final cooling (50 °C, 30 s). Relative gene expression levels were calculated using 2^−ΔΔCT^ method. The primer sequences are outlined in Table 1.

**TABLE 1:** Primer sequences for quantitative real-time PCR.

Target (GenBank accession no.)	Primers
c-Fos (NM_010234.3)	F: ACA​GCC​TTT​CCT​ACT​ACC​ATT​CC
	R: GGC​ACT​AGA​GAC​GGA​CAG​ATC
CTSK (NM_007802.4)	F: AGC​AGA​ACG​GAG​GCA​TTG​AC
	R: ATT​TAG​CTG​CCT​TTG​CCG​TG
DC-STAMP (NM 029422.4)	F: TTC​TCG​TGT​CAG​TCT​CCT​TCT​ACC
	R: TTT​CCC​GTC​AGC​CTC​TCT​CAA
OC-STAMP (NM_029021.1)	F: CCA​CTG​TCC​CAA​TCA​CAC​TCA
	R: GTG​GTA​GAT​GAC​AGT​CGT​GGG
MMP-9 (NM_013599.5)	F: GCC​CTG​GAA​CTC​ACA​CGA​CA
	R: TTG​GAA​ACT​CAC​ACG​CCA​GAA​G
NFATc1 (NM_001164109.1)	F: AGT​CTC​ACC​ACA​GGG​CTC​AC
	R: TCA​GCC​GTC​CCA​ATG​AAC​AG
GAPDH (XM_036165840.1)	F: TGT​GTC​CGT​CGT​GGA​TCT​GA
	R: TTG​CTG​TTG​AAG​TCG​CAG​GAG

F: forward primer; R: reverse primer; c-Fos: Fos proto-oncogene, AP-1 transcription factor subunit; CTSK: cathepsin K; DC-STAMP: dendritic cell–specific transmembrane protein; OC-STAMP: osteoclast stimulatory transmembrane protein; MMP-9: matrix metallopeptidase 9; NFATc1, nuclear factor of activated T-cells 1; GADPH: glyceraldehyde-3-phosphate dehydrogenase.

### 2.11 Intracellular reactive oxygen species detection

Intracellular reactive oxygen species (ROS) levels were assessed using the H_2_DCFDA fluorescent probe (Beyotime, S0033S) as previously described (Wang et al., 2025). Osteoclast precursors were pretreated with or without MR for 24 h. The experimental groups included a control group treated with RANKL (30 ng/mL) and an MR group treated with RANKL (30 ng/mL) and MR (1 mM). Cells were then treated with DCFH-DA (10 μM) at 37 °C for 20 min in the dark. Fluorescence images were captured from six random fields per well using an EVOS M7000 imaging system. Fluorescence intensity was quantified with ImageJ after background subtraction and threshold normalization.

### 2.12 Ovariectomy-induced osteoporosis model

An ovariectomy (OVX)-induced osteoporosis model was established in mice to evaluate the *in vivo* effects of MR on bone loss. A total of fifteen 8-week-old female C57BL/6J mice were randomly divided into three groups: sham, OVX and OVX + MR. Under aseptic conditions and intraperitoneal anesthesia, mice in the OVX and OVX + MR groups underwent bilateral ovariectomy through dorsal approach, whereas mice in the sham group underwent the same surgical procedure without removal of the ovaries. After a 7-day recovery period, mice in the OVX + MR group received intraperitoneal injections of MR every other day at a dose of 25 mg/kg, while OVX and sham groups received equivalent volumes of saline. After 4 weeks of treatment, all mice were humanely euthanized by cervical dislocation for tissue collection.

### 2.13 Microcomputed tomography (μCT) analysis

The left femora were fixed in 4% PFA for 24 h. The distal metaphysis of each femur was scanned using a Quantum GX2 system (PerkinElmer) under standardized parameters: 114 μA current, 70 kVp tube voltage, and high-resolution acquisition mode with a scan duration of 14 min. The acquired images were reconstructed using Analyze 12.0 software, and trabecular microarchitecture parameters were quantified by measuring bone volume (BV, mm^3^), bone volume fraction (BV/TV, %), trabecular thickness (Tb.Th, mm), trabecular separation (Tb.Sp, mm), and mean bone mineral density of trabecular tissue (BMD, mg/cm^3^).

### 2.14 Histological analysis

The right femora were fixed in 4% PFA for 24 h, decalcified, embedded in paraffin, and sectioned at a thickness of 5 μm. The tissue sections were stained with H&E (hematoxylin and eosin) for histomorphometric evaluation. The TRAP staining solution for bone tissue sections was prepared using the same procedure described above. TRAP-positive osteoclasts were then observed under a light microscope and quantified.

### 2.15 Statistical analysis

All experiments were conducted at least in triplicate, and results are presented as mean ± standard deviation (SD). Student’s t-test was implemented for two-group comparison, and one-way analysis of variance (ANOVA) for multiple-group comparisons. Statistical analyses were conducted with GraphPad Prism (v. 9.5.0), with significance defined as *P* < 0.05.

## 3 Results

### 3.1 Identification of potential targets of MR against osteoporosis

**FIGURE 1:**
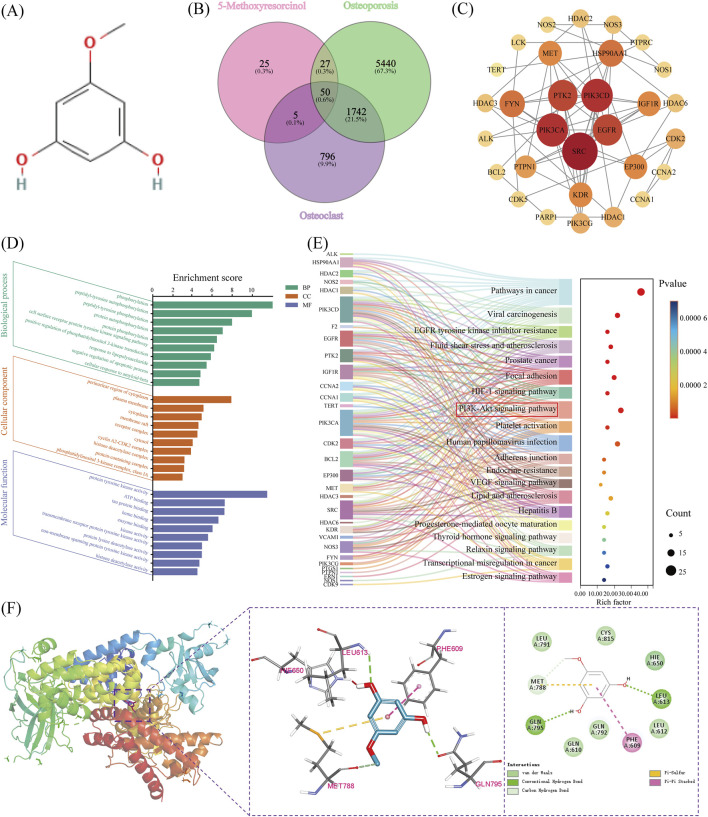
Network pharmacology-based target prediction and molecular docking of 5-methoxyresorcinol. **(A)** Molecular structure of 5-methoxyresorcinol (MR). **(B)** Venn diagram showing overlapping gene targets between MR and osteoporosis/osteoclasts. **(C)** Protein-protein interaction (PPI) network of the 50 overlapping targets. **(D)** Gene Ontology (GO) enrichment analysis (top 10 terms) of the 50 overlapping targets (BP: Biological Process, CC: Cellular Component, and MF: Molecular Function). **(E)** Kyoto Encyclopedia of Genes and Genomes (KEGG) pathway enrichment analysis (top 20 pathways) of the 50 overlapping targets. **(F)** Molecular docking results illustrating the interaction between MR and PI3K.

To investigate how MR alleviates osteoporosis, we applied a network pharmacology approach. Based on the molecular structure of MR, 107 potential gene targets were identified using the Swiss Target Prediction database (Figure 1A; Supplementary Table S1). From the GeneCards database, 7,259 and 2,593 targets related to osteoporosis pathogenesis were retrieve using the keywords *“osteoporosis”* and *“osteoclast,”* respectively (Supplementary Tables S2, S3). Intersection analysis using Venny platform identified 50 overlapping targets between MR and osteoporosis related genes (Figure 1B; Supplementary Table S4). A PPI network was subsequently constructed for these 50 shared targets using the STRING database (Figure 1C). Based on degree values, SRC, PIK3CA, and PIK3CD were identified as the top three hub genes. Given the critical role of PI3K in osteoclast proliferation and differentiation (Lu et al., 2024), PI3K was selected as a core target protein through which MR exerts its therapeutic effects. To further explore the potential mechanisms of MR, KEGG and GO enrichment analyses were performed. GO analysis indicated that the overlapping targets were mainly enriched in protein phosphorylation related biological processes, whereas KEGG analysis highlighted significant enrichment of the PI3K/AKT signaling pathway (Figures 1D,E). In summary, these findings suggest that MR may exert anti-osteoporotic effects through modulation of the PI3K/AKT signaling pathway. The bioinformatics analysis was completed in December 2024.

### 3.2 Molecular docking

To further validate the interactions predicted by the network pharmacology, molecular docking was performed between MR and PI3K. The docking results showed that MR formed hydrogen bonds with GLN-795 and LEU-613, π–sulfur and π–π stacking interactions with MET-788 and PHE-629, and van der Waals interactions with residues, including LEU-791, LEU-612, and GLN-610 (Figure 1F). The calculated binding energy was −5.2 kcal/mol, indicating a stable interaction.

### 3.3 Molecular dynamics simulation

**FIGURE 2:**
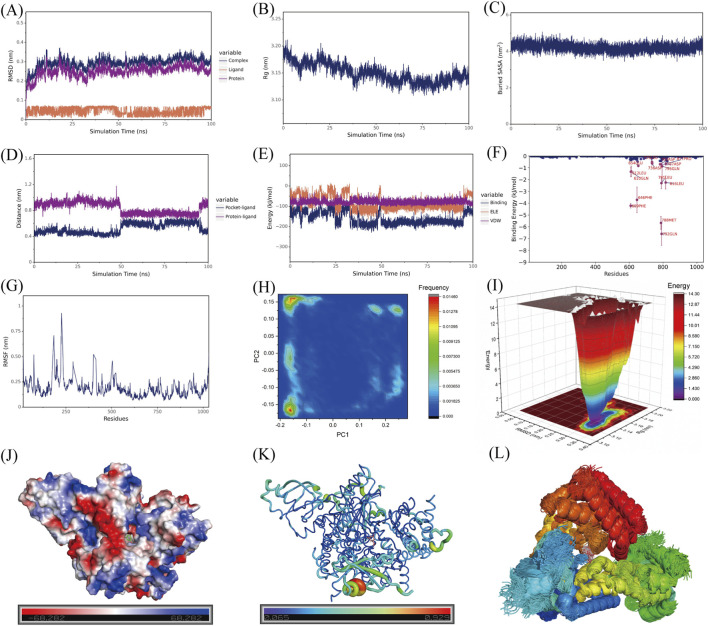
100 ns Molecular Dynamics Simulation of 5-methoxyresorcinol with PI3K. **(A)** Root-mean-square deviation (RMSD) of the 5-methoxyresorcinol (MR) molecule, PI3K protein, and the MR–PI3K complex. **(B)** Radius of gyration (Rg) of the MR–PI3K complex. **(C)** Buried solvent-accessible surface area (Buried SASA) between MR and PI3K. **(D)** Variation in the distance between MR and the binding site of PI3K. **(E)** Time-dependent changes in electrostatic (ELE) and van der Waals (VDW) interaction energies during the simulation. **(F)** Per-residue energy contribution to the binding free energy. **(G)** Root-mean-square fluctuation (RMSF) of PI3K. **(H)** Principal component analysis (PCA) of the ligand trajectory within the complex. **(I)** Free energy landscape (FEL) derived from the molecular simulation. **(J)** Surface electrostatic potential of the binding pocket. **(K)** B-factor plot generated based on RMSF values, illustrating the flexibility of protein residues around the bound ligand. **(L)** Superposition of representative conformational snapshots extracted from the simulation trajectory.

To further evaluate the stability of the MR–PI3K interaction, a 100 ns molecular dynamics simulation was performed for the MR-PI3K complex. The system remained stable throughout the simulation, as evidenced by convergence of RMSD (root mean square deviation) and Rg (radius of gyration) values (Figures 2A,B). The buried solvent accessible surface area (Buried SASA) also gradually plateaued, indicating that the contact interface between MR and PI3K became stable over time (Figure 2C). The distance between MR and PI3K centroid, as well as the distance between MR and the binding pocket, remained stable (Figure 2D). Energy decomposition analysis showed that van der Waals interactions contributed predominantly to complex formation, with additional contributions from electrostatic and hydrophobic interactions. The van der Waals and electrostatic interaction energies of the complex remained stable (Figure 2E). Key residues mediating binding included GLN-792 and MET-788 (Figure 2F). Principal component analysis (PCA) revealed a dominant conformational cluster (Figure 2H) and the free energy landscape (FEL) showed that this conformation resides in a low-energy basin (Figure 2I), indicating a favorable and stable binding state. The ΔE_MMPBSA_ binding free energy was −45.173 ± 2.146 kJ/mol, confirming a strong binding affinity. Structural visualization further showed that MR was accommodated within a groove of the protein, where the binding interface was predominantly electroneutral and negatively charged, a feature that may facilitate electrostatic interactions and hydrogen bonding (Figure 2J). Furthermore, the B-factor profile derived from RMSF values indicated low flexibility of the residues surrounding the MR binding site, further supporting the stability of the MR–PI3K complex (Figures 2G,K). Consistently, the high positional overlap of MR throughout the simulation trajectory indicated persistent occupancy of the binding pocket (Figure 2L). Collectively, these results support the formation of a stable MR–PI3K complex andprovide further evidence that MR may act as a potential modulator of PI3K signaling.

### 3.4 MR attenuates RANKL-Induced osteoclastogenesis

**FIGURE 3:**
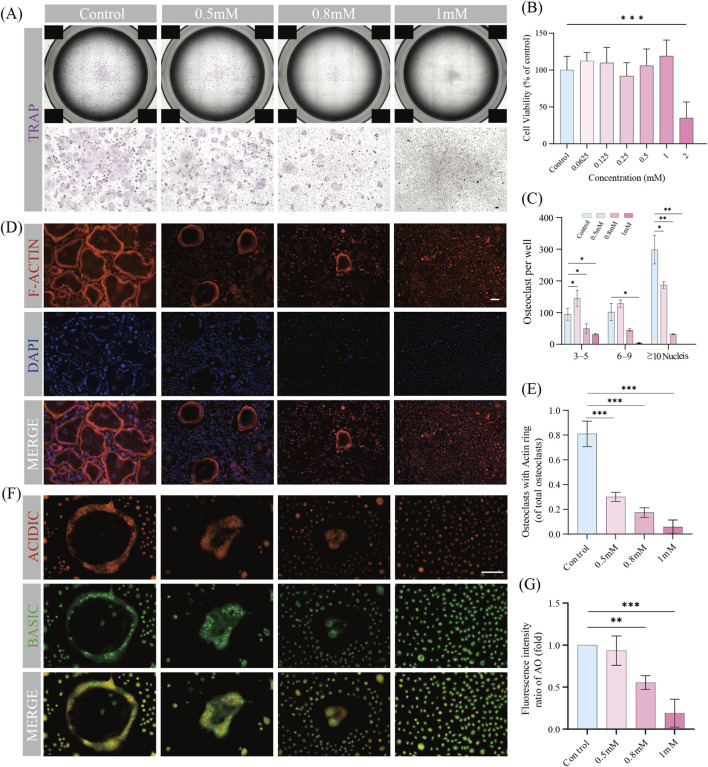
Effects of 5-methoxyresorcinol on osteoclast differentiation and function. **(A)** TRAP staining of osteoclasts treated with gradient concentrations of 5-methoxyresorcinol (MR) (0, 0.5 mM, 0.8 mM, 1 mM). **(B)** Cytotoxicity assay of MR. **(C)** Quantitative analysis of TRAP-positive cells categorized by the number of nuclei per cell (3–5, 6–9, ≥10 nuclei) under different MR concentrations. **(D)** F-actin ring staining of osteoclasts with gradient concentrations of MR (0, 0.5 mM, 0.8 mM, 1 mM). **(E)** Proportion of osteoclasts with intact actin rings relative to the total number of osteoclasts at varying MR concentrations. **(F)** Acridine orange staining with gradient concentrations of MR (0, 0.5 mM, 0.8 mM, 1 mM). **(G)** Quantitative analysis of the red-to-green fluorescence intensity ratio under different MR concentrations. The data of Figure G represent the relative fold change compared to the control group, which was defined as 1. Data are shown as means ± SD (n = 3, *p < 0.05, **p < 0.01, and ***p < 0.001). Scale bar = 100 μm.

The cytotoxicity of MR toward BMMs was assessed using the CCK-8 assay. As indicated in Figure 3B, no significant toxicity was observed at concentrations ≤1 mM. Based on this result, subsequent experiments were performed using MR at concentrations of 0.5, 0.8, and 1 mM. The effect of MR on RANKL-induced osteoclast differentiation was then examined by TRAP staining. In the control group, numerous TRAP-positive multinucleated osteoclasts were observed, whereas MR treatment markedly suppressed osteoclast formation in a dose-dependent manner (Figure 3A). Quantitative analysis further showed that MR significantly reduced the number of osteoclasts containing 3–5, 6–9, and ≥10 nuclei (Figure 3C), indicating an inhibitory effect on osteoclast maturation. To further determine whether MR affected osteoclast function, acidified vesicle formation and F-actin ring assembly were examined. Over 80% of osteoclasts in the control group formed intact F-actin rings, whereas this proportion decreased to less than 10% following treatment with 1 mM MR (Figures 3D,E). AO staining showed a marked reduction red-to-green fluorescence ratio after MR treatment, indicating impaired cidification activity (Figures 3F,G). Together, these findings demonstrate that MR suppresses both the differentiation and functional activity of osteoclasts induced by RANKL.

### 3.5 MR inhibits osteoclastogenesis via the PI3K-AKT signaling axis

**FIGURE 4:**
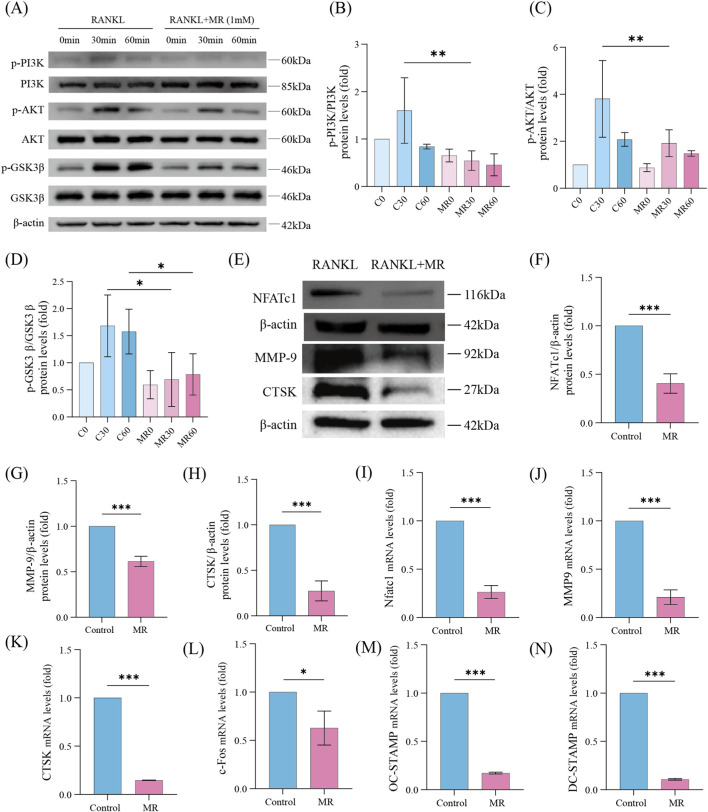
5-methoxyresorcinol inhibits osteoclast differentiation and function by suppressing the PI3K-AKT signaling pathway. **(A)** Protein expression levels of p-PI3K, PI3K, p-AKT, AKT, p-GSK3β and GSK3β in osteoclasts treated with 1 mM MR for 0, 30, and 60 min. **(B–D)** Quantitative analysis of the p-PI3K/PI3K ratio **(B)**, p-AKT/AKT ratio **(C)**, and p-GSK3β/GSK3β ratio **(D)** following treatment with 1 mM MR for 0, 30, and 60 min. **(E)** Protein expression levels of NFATc1, MMP-9 and CTSK in osteoclasts on days 3–4 with RANKL. **(F)** Quantitative analysis of the NFATc1 protein expression levels. **(G)** Quantitative analysis of the MMP-9 protein expression levels. **(H)** Quantitative analysis of the CTSK protein expression levels. **(I–N)** Quantitative analysis of mRNA expression levels of NFATc1 **(I)**, MMP9 **(J)**, CTSK **(H)**, c-Fos **(L)**, OC-STAMP **(M)**, and DC-STAMP **(N)** on days 4–5 after treatment with 1 mM MR. Data represent the relative fold change compared to the control group, which was defined as 1. Data are shown as means ± SD (n = 3, *p < 0.05, **p < 0.01, and ***p < 0.001).

Network pharmacology identified the PI3K-AKT axis as a primary target of MR, consistent with the established role of this pathway in osteoclast differentiation and activation (Lu et al., 2024; Li J. et al., 2024). To verify this prediction, the activation status of the PI3K/AKT pathway was examined in RANKL-stimulated osteoclast precursors. RANKL stimulation rapidly activated PI3K, AKT, and GSK3β phosphorylation, whereas MR treatment significantly suppressed phosphorylation of all three proteins (Figures 4A–D). In addition, MR also reduced the expression of NFATc1, a key transcription factor required for osteoclastogenesis (Figures 4E,F). Western blot analysis further showed that MR markedly decreased the protein expression levels of CTSK and MMP-9 (Figures 4G,H). Next, we evaluated the effect of MR on osteoclast signature gene expression. As shown in Figures 4I–N, MR treatment significantly downregulated the expression of genes essential for osteoclast differentiation (c-FOS, NFATc1, DC-STAMP, and OC-STAMP) as well as key functional markers of bone resorption (CTSK and MMP-9). Collectively, these results indicate that MR inhibits osteoclastogenesis at least in part through suppression of the PI3K/AKT signaling pathway.

### 3.6 AKT agonist SC79 reverses the MR mediated inhibition of osteoclastogenesis

**FIGURE 5:**
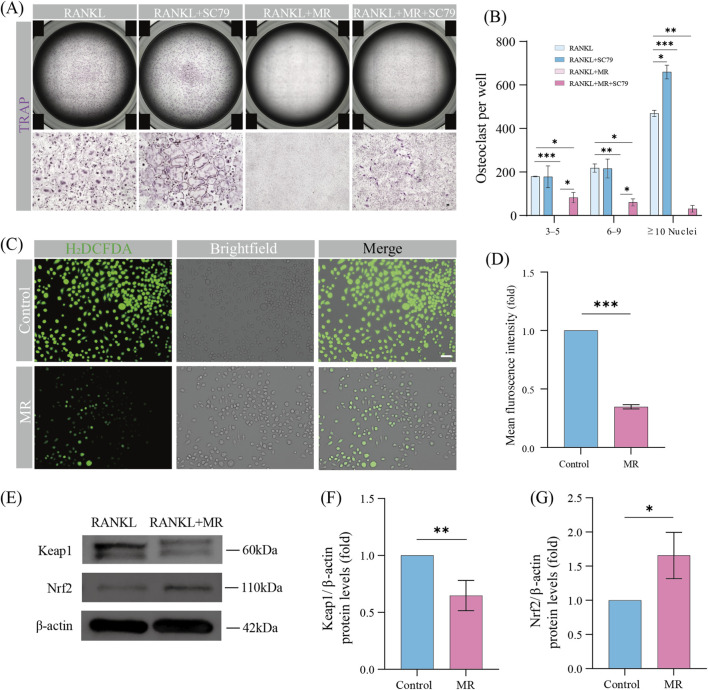
SC79 counteracts MR induced inhibition of osteoclast differentiation; MR promotes intracellular ROS scavenging through Keap1/Nrf2 signaling. **(A)** Representative images of TRAP staining showing osteoclasts treated with MR (1 mM) and SC79 (2 μg/mL). Scale bar = 100 μm. **(B)** Quantitative analysis of TRAP-positive cells categorized by the number of nuclei per cell (3–5, 6–9, ≥10 nuclei) under MR (1 mM) and SC79 (2 μg/mL). **(C)** Intracellular ROS levels in osteoclasts after treatment with 1 mM MR. Scale bar = 100 μm. **(D)** Quantitative analysis of intracellular ROS levels following treatment with 1 mM MR. **(E)** Protein expression levels of Keap1 and Nrf2 in osteoclasts on days 3–4 with RANKL. **(F)** Quantitative analysis of the Keap1 protein expression levels. **(G)** Quantitative analysis of the Nrf2 protein expression levels. Data represent the relative fold change compared to the control group, which was defined as 1. Data are shown as means ± SD (n = 3, *p < 0.05, **p < 0.01, and ***p < 0.001).

To further determine whether the PI3K/AKT pathway is involved in the anti-osteoclastogenic effect of MR, we performed rescue experiments using SC79, a specific AKT agonist (Chen et al., 2018). Based on established protocols, SC79 was added to the osteoclast culture systemat a concentration of 2 μg/mL (Bai et al., 2026). By day 4 of RANKL-induced differentiation, the SC79 markedly promoted osteoclast differentiation compared to the control group (Figures 5A,B). It confirmed the role of AKT activation in driving this process, which aligns with prior literature (Bai et al., 2026). Notably, the inhibitory effect of MR on osteoclast differentiation was partially reversed by co-treatment with SC79, as evidenced by the reappearance of osteoclasts in the MR + SC79 group. Taken together, these results further support the conclusion that MR inhibits osteoclast differentiation through the PI3K/AKT pathway downstream of RANKL signaling.

### 3.7 MR suppresses RANKL-Induced ROS generation in osteoclast precursors

Accumulating evidence suggests that ROS drive osteoclast proliferation and differentiation by activating signaling pathways like MAPK, PI3K–AKT, and NF-κB (Meng et al., 2021; Li H et al., 2024). Given that MR has been reported to exert antioxidant and anti-inflammatory effects, we next examined whether MR could regulate intracellular ROS levels in osteoclast precursors. H_2_DCFDA fluorescence assay showed that RANKL stimulation (30 ng/mL) markedly increased intracellular ROS accumulation in osteoclast precursors, whereas MR treatment dose-dependently suppressed this effect (Figures 5C,D).

The Keap1/Nrf2 signaling pathway is a critical regulator of cellular redox homeostasis (Adinolfi et al., 2023). Increased ROS levels trigger the dissociation of Nrf2 from its inhibitor Keap1, leading to Nrf2 nuclear translocation and subsequent activation of the downstream antioxidant defense system (Chi et al., 2025). To further elucidate the underlying mechanism of ROS clearance by MR, the protein expression levels of Keap1 and Nrf2 were assessed. MR treatment significantly decreased Keap1 expression while increasing Nrf2 protein levels (Figures 5E–G). Taken together, these results suggest that MR suppresses intracellular ROS accumulation, through the Keap1/Nrf2 signaling pathway.

### 3.8 MR alleviates osteoporotic bone loss in mice

**FIGURE 6:**
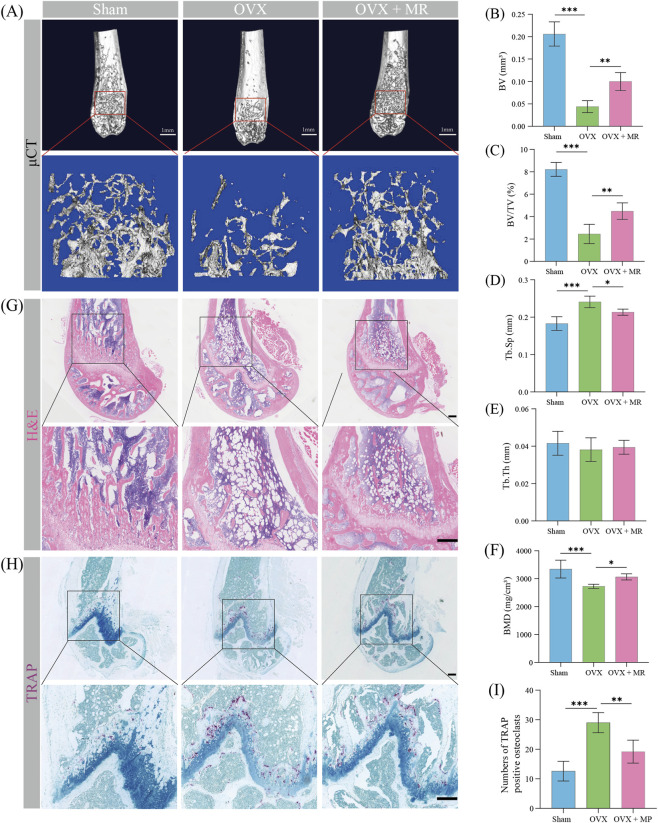
5-methoxyresorcinol ameliorates bone loss in OVX mice. **(A)** 3D micro-computed tomography (μCT) reconstruction images of the femur in OVX mice. Scale bar = 1 mm. **(B–F)** Quantitative μCT analysis of the distal femur: **(B)** bone volume (BV), **(C)** bone volume fraction (BV/TV), **(D)** trabecular separation (Tb.Sp), **(E)** trabecular thickness (Tb.Th), and **(F)** mean bone mineral density of trabecular tissue (BMD, mg/cm^3^). **(G)** Hematoxylin and eosin (H&E) staining of the distal femur. Scale bar = 200 μm. **(H)** TRAP staining of the distal femur. **(I)** Quantitative analysis of TRAP positive osteoclasts. Scale bar = 200 μm. Data are shown as means ± SD (n = 5, *p < 0.05, **p < 0.01, and ***p < 0.001).

To further evaluate the anti-osteoporotic effect of MR *in vivo*, OVX-induced osteoporosis model was established to mimic postmenopausal bone loss. Three-dimensional μCT reconstructions showed that, *versus* the sham group, OVX mice showed marked trabecular bone loss and deterioration of bone microarchitecture, whereas MR treatment effectively prevented these pathological changes (Figure 6A). Quantitative μCT analysis showed that the OVX group displayed significantly lower BV and BV/TV, together with increased Tb. Sp, compared with the sham group (Figures 6B–D). In contrast, MR-treated OVX mice displayed higher BV and BV/TV and a significant reduction in Tb. Sp compared with the OVX group. No significant differences were observed in Tb.Th among the three groups (Figure 6E). Moreover, BMD was significantly reduced in OVX mice but was markedly restored by MR treatment (Figure 6F).

Histological evaluation with H&E staining further supported the μCT findings. Compared with the sham group, OVX mice exhibited sparse and disorganized trabeculae accompanied by obvious bone loss. In comparison, MR treatment partially preserved trabecular continuity and bone structure in OVX mice (Figure 6G). In addition, TRAP staining showed that the number of osteoclasts was markedly increased in the OVX group compared with the sham group, whereas MR treatment significantly reduced osteoclast number in OVX mice (Figures 6H,I). Collectively, these results indicate that MR effectively attenuates OVX-induced osteoporotic bone loss *in vivo*.

## 4 Discussion

Excessive osteoclast-mediated bone resorption and dysregulated bone remodeling are central pathological features of osteoporosis (Sirikul et al., 2022; Rachner et al., 2011). These changes are particularly pronounced in postmenopausal women, as estrogen deficiency enhances osteoclast formation while inhibiting osteoblast differentiation (Yao et al., 2025; Yang et al., 2022). Accordingly, the identification of agents capable of suppress osteoclast activity has become an important strategy for the prevention and treatment of osteoporosis (Zhang et al., 2020; Yang et al., 2023). In the present study, we systematically investigated the therapeutic potential of MR in osteoporosis and elucidated its underlying mechanisms through network pharmacology, *in vitro* experiments, and *in vivo* validation. Collectively, our findings indicate that MR alleviates osteoporosis by inhibiting RANKL-induced osteoclast differentiation and function through modulation of the PI3K–AKT signaling pathway and intracellular ROS homeostasis.

Natural compounds have attracted increasing attention in the treatment of metabolic bone diseases because of their structural diversity, multitarget properties, and relatively low toxicity. For example, Liu et al. reported that hecogenin activates Nrf2, reduces ROS accumulation, inhibits pyroptosis, and suppresses the NLRP3 inflammasome activation, thereby attenuating osteoclast formation and reducing LPS-induced inflammatory osteolysis (Liu et al., 2024). Similarly, MA, a pentacyclic triterpene acid, has been shown to ameliorate osteoporosis by suppressing RANKL-induced osteoclast differentiation through the MAPK/AP-1 and NF-κB signaling pathways (Li et al., 2011). MR is one of the active ingredients in Pra-Sa-Chang-Dang and our preliminary bioinformatics screening suggests that it may also possess anti-osteoporotic potential.

To the best of our knowledge, this is the first study to integrate network pharmacology with experimental validation to identify potential therapeutic targets of MR in osteoporosis. GO and KEGG enrichment analyses highlighted the PI3K–AKT axis as a key pathway potentially involved in the action of MR. Molecular docking provided preliminary evidence supporting the interaction between MR and PI3K, while molecular dynamics simulations further suggested that MR could form a stable, high-affinity complex with PI3K, characterized by low binding free energy and sustained conformational stability during the 100-ns simulation. Taken together, these findings support MR as a potential agent for the clinical prevention and management of osteoporosis.

Our *in vitro* experiments further validated the anti-osteoclastic activity of MR. The CCK-8 assay showed that MR at concentrations ≤1 mM exhibited no cytotoxicity toward BMMs, supporting the suitability of this concentration range for subsequent functional experiments. TRAP staining showed that MR dose-dependently suppressed RANKL-induced osteoclast formation, significantly reducing the number of multinucleated (≥3 nuclei) TRAP^+^ cells. Mature osteoclasts are characterized by specialized cytoskeletal structures composed of columnar F-actin cores arranged into ring-like structures (Chen et al., 2023), which are critical for adhesion and motility (Marchisio et al., 1984). Acidified vesicles derived from the cytoplasm are subsequently secreted into the sealed resorption lacuna enclosed by the F-actin ring, thereby enabling degradation of the mineralized matrix (Pierzyńska-Mach et al., 2014; Cui et al., 2022). In the present study, MR markedly suppressed osteoclast function by inhibiting the formation of F-actin ring and reducing acidified vesicle secretion, as shown by F-actin and AO staining. These phenotypic changes were accompanied by molecular alterations. MR down regulated osteoclast-specific genes, including NFATc1 and c-FOS (master regulators of differentiation), DC-STAMP and OC-STAMP (mediators of cell fusion), and CTSK and MMP-9 (key enzymes in bone resorption). Overall, these findings confirm that MR inhibits osteoclastogenesis and function at both cellular and molecular levels.

**FIGURE 7:**
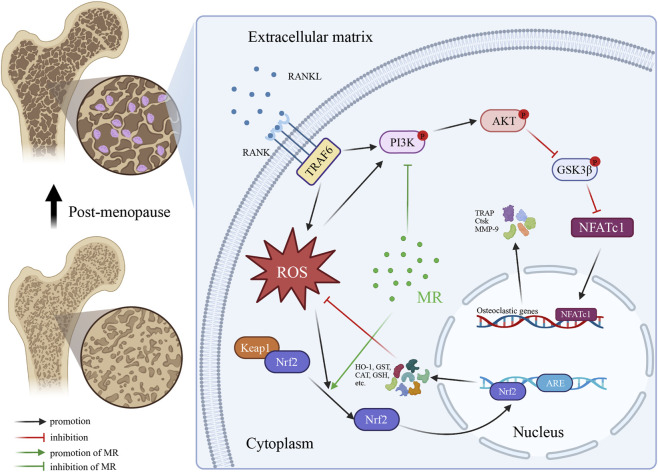
5-methoxyresorcinol inhibits osteoclast differentiation and function via the PI3K–AKT–GSK3β axis and intracellular ROS scavenging.

Mechanistically, our network pharmacology analysis identified 50 overlapping targets between MR and osteoporosis, with significant enrichment in the PI3K–AKT signaling pathway. GSK3β, a well-known downstream target of AKT, transduces RANKL-induced signals to enhance the nuclear export of NFAT proteins (Moon et al., 2012; Cross et al., 1995). Consistent with these predictions, our *in vitro* experiments showed that MR markedly inhibited RANKL-induced phosphorylation of PI3K, AKT, and GSK3β, thereby inhibiting activation of the PI3K–AKT axis. Simultaneously, MR reduced RANKL-induced intracellular ROS accumulation in osteoclast precursors, consistent with previous reports that ROS activate PI3K–AKT signaling to promote osteoclastogenesis (Chen et al., 2022). Based on these findings, we propose that MR exerts its anti-osteoclastogenic effects through a dual regulatory mechanism: directly inhibiting PI3K–AKT pathway phosphorylation to suppress NFATc1-mediated gene expression, while also reducing ROS accumulation and thereby attenuating ROS-dependent activation of PI3K–AKT (Figure 7). This coordinated regulation amplifies MR’s efficiency in disrupting osteoclastogenesis. *In vivo* validation using an OVX-induced osteoporosis model further supported the anti-osteoporotic potential of MR. μCT analysis showed that MR significantly improved trabecular bone microarchitecture in OVX mice, as reflected by increased BV/TV, and BMD together with reduced Tb. Sp. H&E staining provided complementary evidence, showing that MR partially preserved trabecular integrity and bone structure. In addition, TRAP staining demonstrated that MR reduced osteoclast number in bone tissue, further supporting its inhibitory effect on osteoclastogenesis *in vivo*. These *in vivo* findings were consistent with the *in vitro* results and indicatethat MR can effectively attenuate estrogen deficiency-induced bone loss. Taken together, the concordance between the *in vitro* and *in vivo* experiments strengthens the overall robustness of our findings and supports the therapeutic potential of MR in osteoporosis.

Despite these promising findings, several limitations of the present study should be acknowledged. First, although our data suggest that both PI3K/AKT signaling and ROS regulation are involved in the action of MR, the causal relationship between these two processes remains to be fully clarified. Future studies employing specific pathway inhibitors, antioxidant interventions, or gene-silencing approaches will be needed to dissect their mechanistic interplay more precisely. Second, because the OVX model primarily reflects postmenopausal osteoporosis, the efficacy of MR in other forms of osteoporosis (e.g., glucocorticoid-induced or age-related osteoporosis) and in humans remains to be evaluated. Finally, long-term toxicity and further pharmacokinetic studies are required before MR can be considered for translational or clinical application.

## 5 Conclusion

In conclusion, this study demonstrates that MR attenuates osteoporosis by inhibiting RANKL-induced osteoclast differentiation and function through regulation of the PI3K–AKT–GSK3β signaling cascade and intracellular ROS homeostasis. These findings not only provide new insights into the anti-osteoporotic mechanism of MR but also support its potential as a promising therapeutic candidate for osteoporosis.

## Data Availability

The original contributions presented in the study are included in the article/Supplementary Material, further inquiries can be directed to the corresponding author.
