# Supplementary material for: 5-methoxyresorcinol mitigates postmenopausal osteoporosis through regulation of the PI3K–AKT–GSK3β signaling pathway and ROS homeostasis
Source: Front Pharmacol. 2026 May 18;17:1734275. doi: 10.3389/fphar.2026.1734275 (PMC13222808; doi:10.3389/fphar.2026.1734275)
Supplement: Supplementary file 1 [file Supplementaryfile1.docx]

**Figure S1.** Full membrane images of all Western blotting in Figure 4A. (A) p-PI3K; (C) PI3K; (C) β-actin.

**Figure S2.** Full membrane images of all Western blotting in Figure 4A. (A) p-AKT; (B) AKT; (C) P-GSK3β; (D) GSK3β; (E) β-actin.

**Figure S3.** Full membrane images of all Western blotting in Figure 4E. (A) MMP-9; (B) β-actin.

**Figure S4.** Full membrane images of all Western blotting in Figure 4E. (A) CTSK; (B) β-actin.

**Figure S5.** Full membrane images of all Western blotting in Figure 4E. (A)NFATc1; (B) β-actin.

**Figure S6.** Full membrane images of all Western blotting in Figure 5E. (A) Keap1; (B) Nrf2; (C) β-actin.


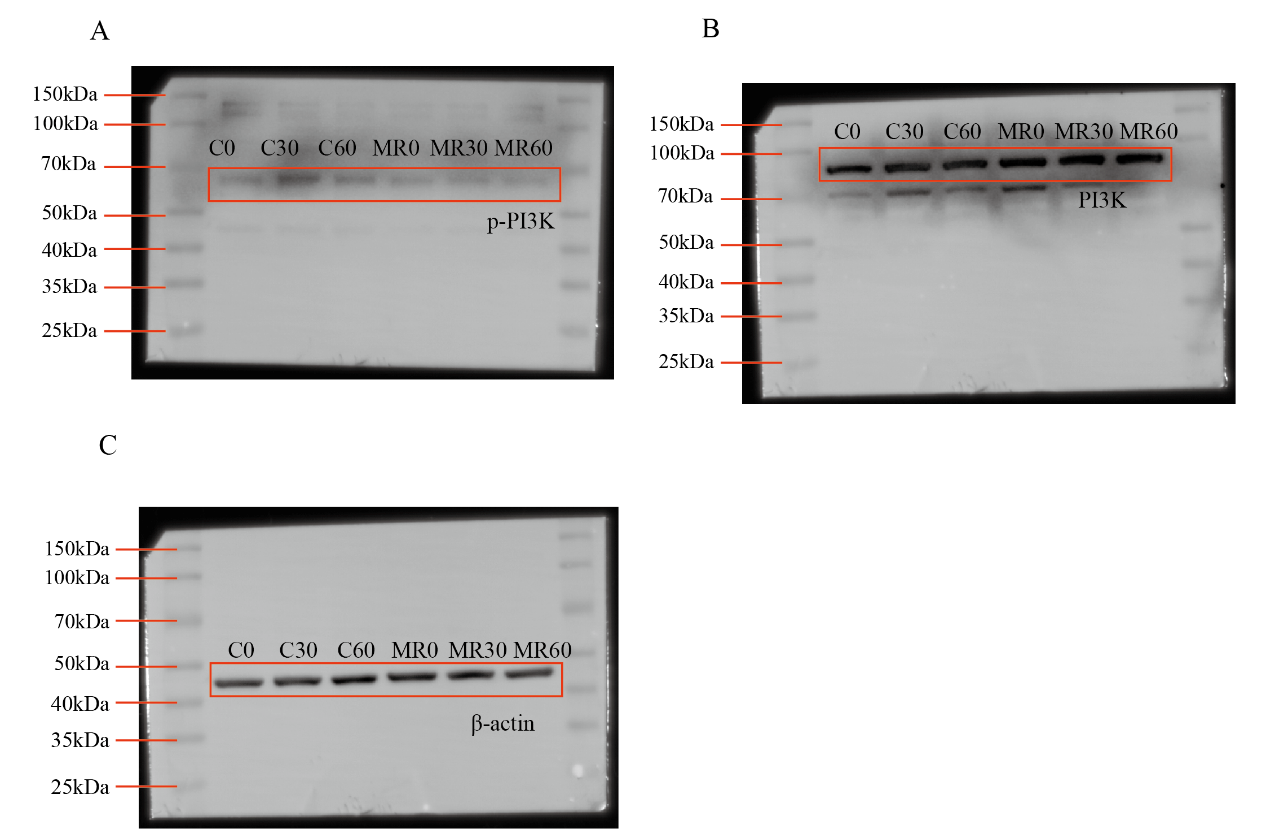


Figure S1. Full membrane images of all Western blotting in Figure 4A. (A) p-PI3K; (C) PI3K; (C) β-actin.


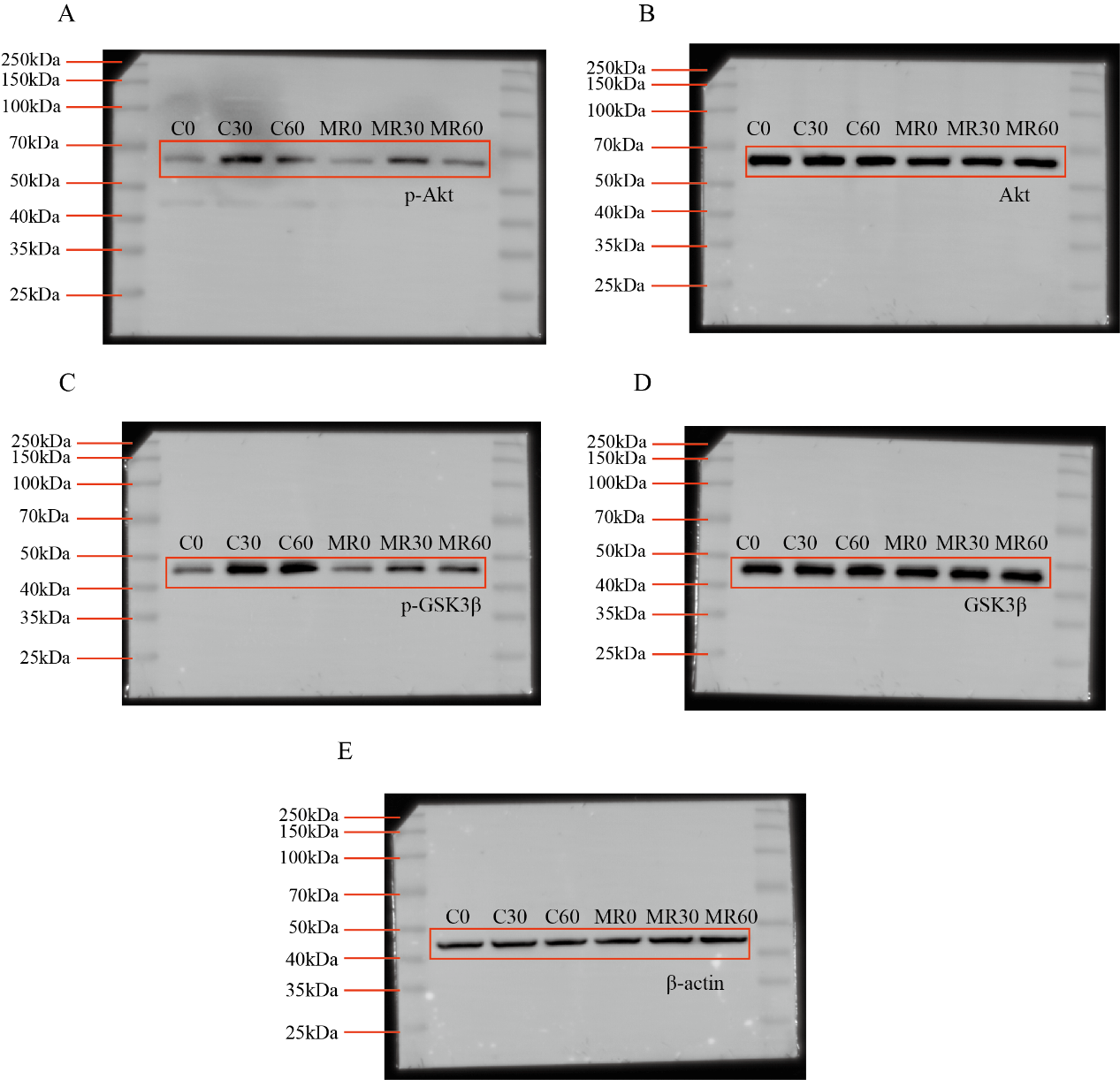


Figure S2. Full membrane images of all Western blotting in Figure 4A. (A) p-AKT; (B) KAT; (C) P-GSK3β; (D) GSK3β; (E) β-actin.

**
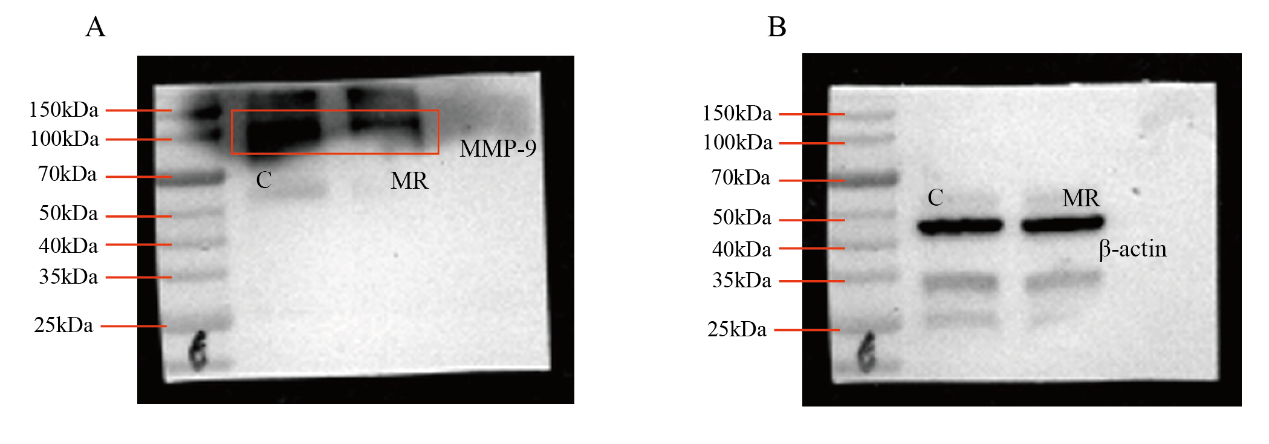
 Figure S3.** Full membrane images of all Western blotting in Figure 4E. (A) MMP-9; (B) β-actin.

**
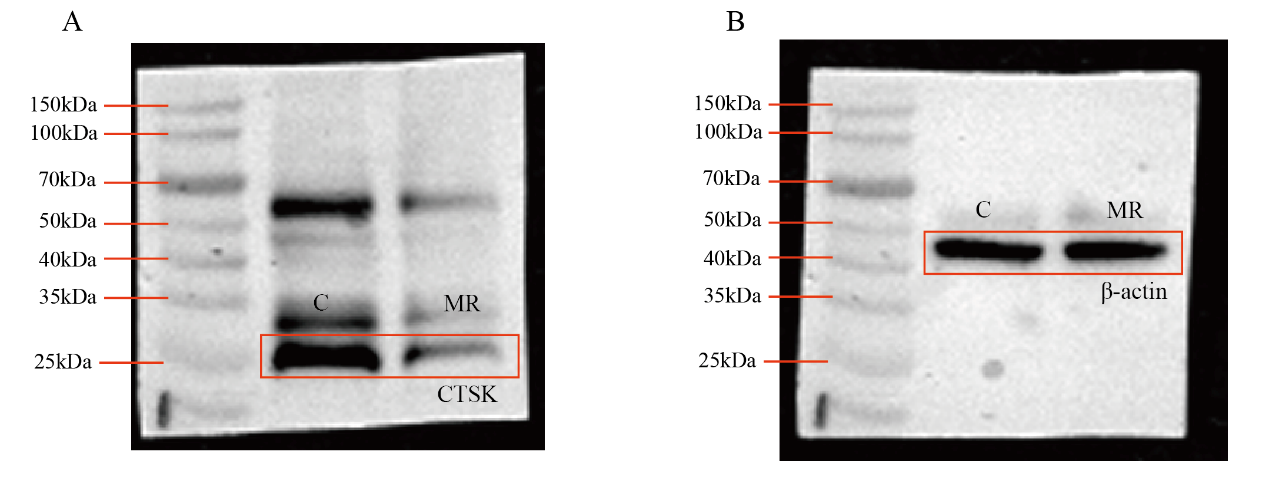
**

**Figure S4.** Full membrane images of all Western blotting in Figure 4E. (A) CTSK; (B) β-actin.


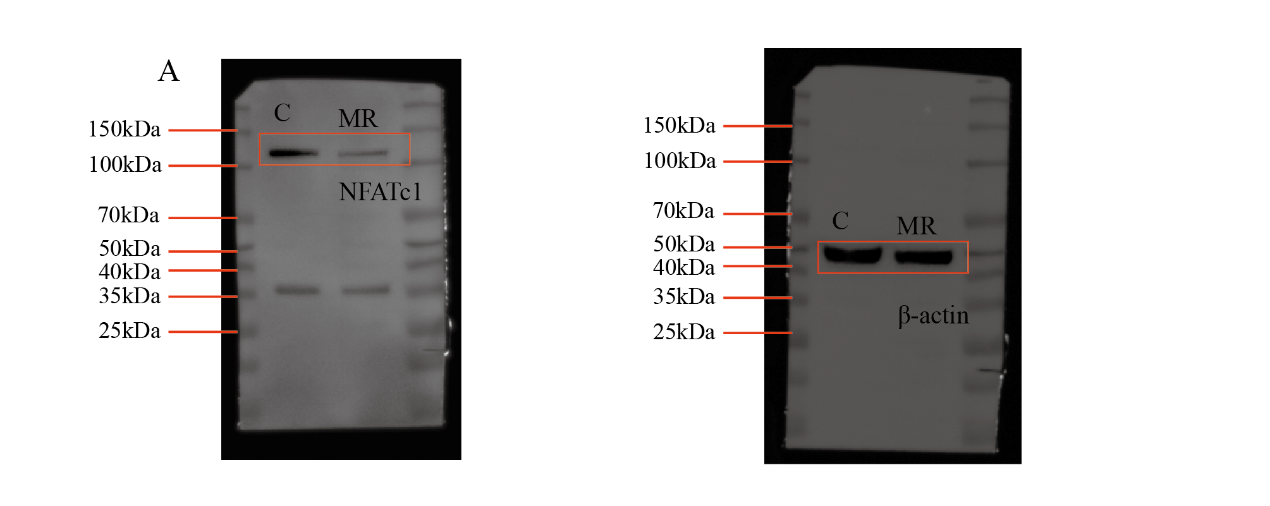


**Figure S5.** Full membrane images of all Western blotting in Figure 4E. (A)NFATc1; (B) β-actin.

**
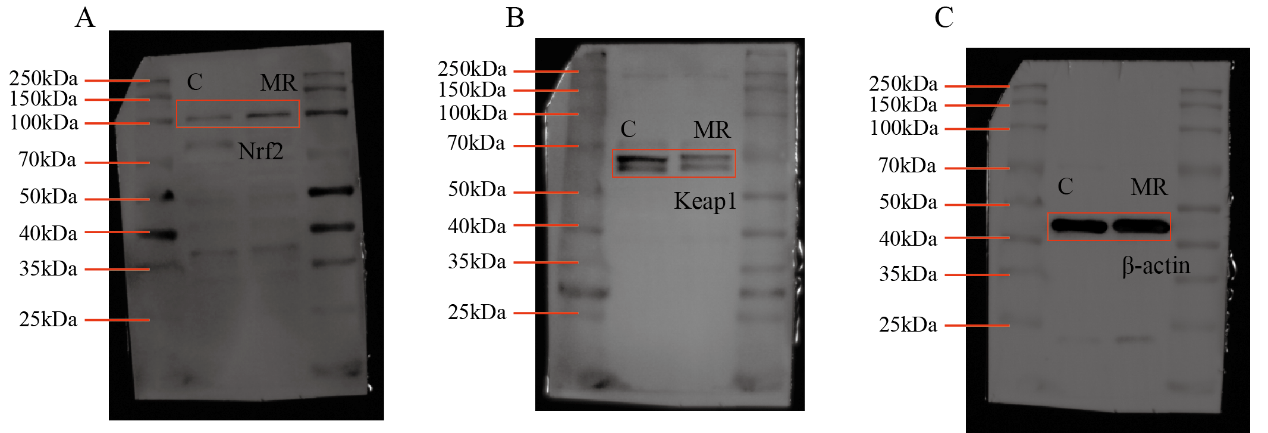
**

**Figure S6.** Full membrane images of all Western blotting in Figure 5E. (A) Keap1; (B) Nrf2; (C) β-actin.
